# Nasopharyngeal flora in children with acute otitis media before and after implementation of 7 valent pneumococcal conjugate vaccine in France

**DOI:** 10.1186/1471-2334-12-52

**Published:** 2012-03-07

**Authors:** Robert Cohen, Edouard Bingen, Corinne Levy, Franck Thollot, Michel Boucherat, Véronique Derkx, Emmanuelle Varon

**Affiliations:** 1 ACTIV, Association Clinique Thérapeutique Infantile du Val de Marne, 27 rue Inkermann, F94100 Saint Maur des Fossés, France; 2 Department of Microbiology, CHI 40 Avenue de Verdun, Créteil, France; 3 Department of Microbiology, Université Denis-Diderot-Paris 7, Robert Debré hospital (AP-HP), 48Bd, Sérurier, 75019 Paris, France; 4 AFPA: Association Française de Pédiatrie Ambulatoire 4 rue Parmentier, F54270, Essey les Nancy, France; 5 National Reference Center for Pneumococci, AP-HP, HEGP 20, rue, Leblanc, F75015 Paris, France

## Abstract

**Background:**

Several studies have investigated the impact of 7-valent pneumococcal conjugate vaccine (PCV7) on pneumococcal (Sp) and staphylococcal (Sa) nasopharyngeal (NP) carriage. Few have investigated the impact on *Haemophilus influenzae *(Hi) and *Moraxella catarrhalis *(Mc) carriage. We aimed to compare the NP carriage rates in young children with acute otitis media (AOM) before and after PCV7 implementation in France.

**Methods:**

Prior to PCV7 implementation, we performed 4 successive randomized trials with NP samples. These studies compared several antibiotic regimens for treating AOM in young children (6 to 30 months). After PCV7 implementation, to assess the impact of the vaccination program on NP flora, young children with AOM were enrolled in a prospective surveillance study. In each study, we obtained an NP sample to analyze the carriage rates of Sp, Hi, Mc and Sa and the factors influencing the carriage. Standardized history and physical examination findings were recorded; the methods used for NP swabs (sampling and cultures) were the same in all studies.

**Results:**

We enrolled 4,405 children (mean age 13.9 months, median 12.8). Among the 2,598 children enrolled after PCV7 implementation, 98.3% were vaccinated with PCV7. In comparing the pre- and post-PCV7 periods, we found a slight but non-significant decrease in carriage rates of pneumococcus (AOR = 0.85 [0.69;1.05]), *H. influenzae *(AOR = 0.89 [0.73;1.09]) and *S. aureus *(AOR = 0.92 [0.70;1.19]). By contrast, the carriage rate of *M. catarrhalis *increased slightly but not significantly between the 2 periods (AOR = 1.08 [0.95;1.2]). Among Sp carriers, the proportion of PCV7 vaccine types decreased from 66.6% to 10.7% (P < 0.001), penicillin intermediate-resistant strains increased from 30.3% to 43.4% (P < 0.001), and penicillin-resistant strains decreased greatly from 22.8% to 3.8% (P < 0.001). The proportion of Hi ß-lactamase-producing strains decreased from 38.6% to 17.1% (P < 0.001).

**Conclusion:**

The carriage rates of otopathogen species (Sp, Hi, Mc) and Sa did not significantly change in children with AOM after PCV7 implementation in France. However, we observed significant changes in carriage rates of PCV7 vaccine serotypes and penicillin non-susceptible Sp.

## Background

The nasopharynx is normally colonized with bacteria such as *Streptococcus viridans*, nonhemolytic streptococci, diphtheroids, and *Neisseria sp*. and potential middle-ear pathogens such as *Streptococcus pneumoniae *(Sp), non-typable *Haemophilus influenzae *(NTHi) and *Moraxella catarrhalis *(Mc) [1]. Furthermore, this ecosystem is the reservoir for *Staphylococcus aureus *(Sa) strains implicated in several infections [2]. The rates of colonization of these potentially pathogenic species are heterogeneously reported in the literature. In fact, in addition to technical procedures of sampling or culture, many other factors may affect colonization rates: daycare attendance; respiratory illness, including acute otitis media (AOM); symptoms of AOM; crowding; season; siblings; and immunization status [3]. Although Sp, NTHi, and Mc are part of the normal nasopharyngeal (NP) flora, increased rate of colonization may identify a subpopulation of children with increased risk of AOM. Indeed, frequency of colonization and AOM is highly correlated (r = 0.37, P < .001) for each pathogen [4]. Furthermore, the disease potential of these pathogens are not identical. Sp is more frequently associated with severe AOM (high fever, otorrhea, mastoïditis), pneumonia and invasive diseases and NTHi with conjunctivitis and recurrent AOM [5,6]. Finally, Mc appears to be the less pathogenic organism [7].

French authorities recommended routine infant immunization with 7-valent pneumococcal conjugate vaccine (PCV7) at age 2, 4 and 12 months for all children [8]. The national PCV7 vaccination coverage has increased slowly and is now relatively high: at least one dose of PCV7 was received by 69% of children less than 2 years old in 2007, 85% in 2008 and 90% in 2009 as compared with 62% in 2006 [9].

Before, during, and after PCV7 implementation, several studies investigated the impact of PCV7 on pneumococcal and staphylococcal carriage [10-12], but few have investigated the impact on NTHi and Mc carriage. Subtle modifications in the equilibrium of NP flora could lead to different etiological profiles of AOM and eventually other respiratory tract infections. We aimed to compare NP carriage of Sp, NTHi, Mc and Sa in young children (6 to 30 months) with AOM before and after PCV7 implementation in France.

## Methods

### Study design

Before the PCV7 implementation, we performed 4 randomized trials on antibiotic treatment for AOM. These studies used standardized protocols (including bacteriological NP samples at enrolment), the same inclusion criteria of AOM [13] and the same age of enrolled children [3,14-16]. Furthermore, the studies involved mostly the same investigators and the same centralized microbiology laboratories. These studies allowed us to identify risk factors for carriage of NP flora bacterial species in children with AOM and to build a protocol for monitoring the impact of the introduction of PCV7 on this ecological niche [3,12,17-20]. Therefore, for the pre-PCV7 implementation period (1993-2000), we used the dataset from the 4 randomized trials [3,14-16], and for the post-PCV7 implementation period (2006- 2009), we used data from an ongoing surveillance study on the impact of PCV7 on NP carriage [12,17-20]. To take into account herd immunity, for the post-PCV7 period, all children were enrolled regardless of their PCV7 vaccination status.

The protocols were approved by the Saint Germain en Laye Hospital Ethics Committee, and written informed consent was obtained from the parents or guardians. The inclusion criteria and definitions were the same during the two periods.

The study populations consisted of children of both sexes; age 4 to 30 months, with newly diagnosed AOM, recruited by pediatricians in pediatric outpatient clinics throughout France. Diagnostic criteria for AOM included the Paradise algorithm for acute suppurative otitis media (effusion + marked redness or marked bulging or moderate redness and bulging) [13]. Patients were not eligible if they had received antibiotic treatment within the 7 days before enrolment. Standardized history and physical examination findings were recorded, and information gathered at study entry included sex, age, daycare modalities, recent antibiotic treatment (within 3 months before enrolment), history of AOM, and clinical symptoms and signs of AOM (thoroughly documented in an identical manner in all trials). The types of antibiotic treatment used by the patients in the studies were most commonly amoxicillinclavulanate and cefpodoxime proxetil.

Daycare modalities were defined according to those most frequently used in France:- Children cared for at home by parents or another person only in the presence of children belonging to the family.- Children cared for by a childcare giver, outside the home, in the presence of other children; a maximum of 3 children is authorized.- Children cared for in a daycare center, which can include many children. In general, one room should be used for the care of no more than 10 children of the same age.

Upon inclusion, the patients were sampled in order to identify bacterial pathogens colonizing the NP niche.

### Microbiologic investigations

The same methods for NP sampling and microbiological testing were used in all studies. Deep NP samples were taken transnasally with use of a flexible, sterile, soft rayon swab tip. After sampling, swabs were immediately inoculated in transport medium (Copan Venturi Transystem, Brescia, Italy), stored at room temperature and sent within 48 hr to laboratories at the National Reference Center for Pneumococci at G. Pompidou European Hospital and to Robert Debré Hospital, in Paris. Swabs were plated within 24 hr onto chocolate agar, *S. aureus *ID agar (SAID; bioMérieux, La Balme Les Grottes, France) 5% horse blood agar. Agar plates were incubated at 37°C for 48 hr under 5% CO_2 _for chocolate and blood agar. Isolates of Sp, Hi, Mc and Sa were identified using colony morphology and conventional methods of determination. *S. pneumoniae *serotyping and antibiotic susceptibility testing were performed at the National Reference Center for Pneumococci. Serotyping was performed using latex particles, which were sensitized with antisera purchased from the Statens Serum Institut (Copenhagen, Denmark). Susceptibility of *S. pneumoniae *isolates to penicillin G was determined from minimal inhibitory concentrations (MICs) by the agar-dilution method. Isolates were classified as penicillin susceptible (MIC ≤ 0.06 μg/ml), penicillin non-susceptible (MIC > 0.12 μg/ml), penicillin intermediate resistant (0.12 ≤ MIC ≤ 1.0 μg/ml), or penicillin resistant (MIC ≥ 2 μg/ml) according to the Clinical and Laboratory Standards Institute [21]. *H. influenzae *isolates underwent capsular serotyping by the slide agglutination method with specific antisera (Phadebact, Boule Diagnostic, Huddinge, Sweden). The production of ß-lactamase was assessed by a chromogenic cephalosporin test (Nitrocefin; Cefinase; Biomerieux, Marcy l'Etoile, France). *H. influenzae *strains were further classified as ampicillin susceptible (MIC ≤ 1 mg/L) or resistant (MIC > 1 mg/L).

### Statistical analyses

Data were double-entered using 4D software (version 6.4), and analysed using Stata SE 9.1 (Stata Corp., College Station, TX, USA) for univariate analysis and multivariate logistic regression (adjusted odds ratios [AORs] and 95% confidence intervals [CI]). For the descriptive statistics, we used mean and median age (months) of children. The Pearson chi-square test was used to compare NP carriage of pneumococci, Hi, Mc and Sa before and after PCV7 implementation. Factors related to NP carriage were identified by univariate analysis (p < 0.20, Pearson chi-square test). These variables, included in multivariate logistic regression models were daycare attendance, recent antibiotic treatment (within 3 months before enrolment), fever ≥ 38.5°C, conjunctivitis, otalgia, and carriage of pneumococci, *H. influenzae, M. catarrhalis *and *S. aureus*. These models were systematically adjusted on the period of study (before and after PCV7 implementation) and on age dichotomized as < 12 and ≥ 12 months. The cut-off of 12 months was chosen for regression model for several reasons: the vaccination schedules differ before and after this age (reflecting the immunity maturation), in many studies, the NP carriage is higher after 12 months, and in one of our studies, young age (< 12 months) predicted penicillin non susceptible pneumococci carriage [19].

## Results

### Study population

The total study population consisted of 4,405 children (mean age 13.9 months, median 12.8): 1,807 were enrolled before PCV7 implementation and 2,598 after. Among the latter group, 98.3% were PCV7 vaccinated. Less than 1% of children had received only one dose of PCV7, and 98.6% were correctly vaccinated for age. Table 1 describes the demographic and clinical characteristics of the 2 populations. Fever (> 38.5°C) and daycare center attendance were more frequent after than before PCV7 implementation. The mean and the median age were slightly higher in the pre-PCV7 period, however, these differences did not exceed 1 month. Otalgia and antibiotic use before enrolment were significantly more frequent before than after PCV7 implementation, with no significant change in type of antibiotic prescribed 3 months before enrolment.

**Table 1:** Demographic and clinical characteristics of children with acute otitis media (AOM) before and after 7-valent pneumococcal conjugate vaccine (PCV7) implementation in France

Child characteristics	Before PCV7 n = 1.807 (%)	After PCV7 n = 2,598 (%)	*p-value*
Male sex'	970 (53.7)	1,376 (53.0)	0.6

Age (months), mean ± SD	14.6 ± 7.6	13.8 ± 5.1	< 0.0001
	
Median	12.8	13.1	

Type of care			< 0.0001
	
Daycare center	436 (24.1)	1032 (39.7)	
	
Child caregiver	530 (29.3)	811 (31.2)	
	
Home	841 (46.6)	754 (29.1)	

Antibiotics 3 months before enrolment	336 (57.2)*	1243 (47.9)	< 0.0001

Conjunctivitis	436 (24.1)	652 (25.1)	0.5

Otalgia	1304 (83.0)**	1945 (75.0)	< 0.0001

Fever (≥ 38.5°C)	671 (37.1)	1551 (60.3)	< 0.0001

* available for 587 patients

** available for 1,571 patients

### NP colonization in the pre-and post-PCV7 periods

**Figure 1:**
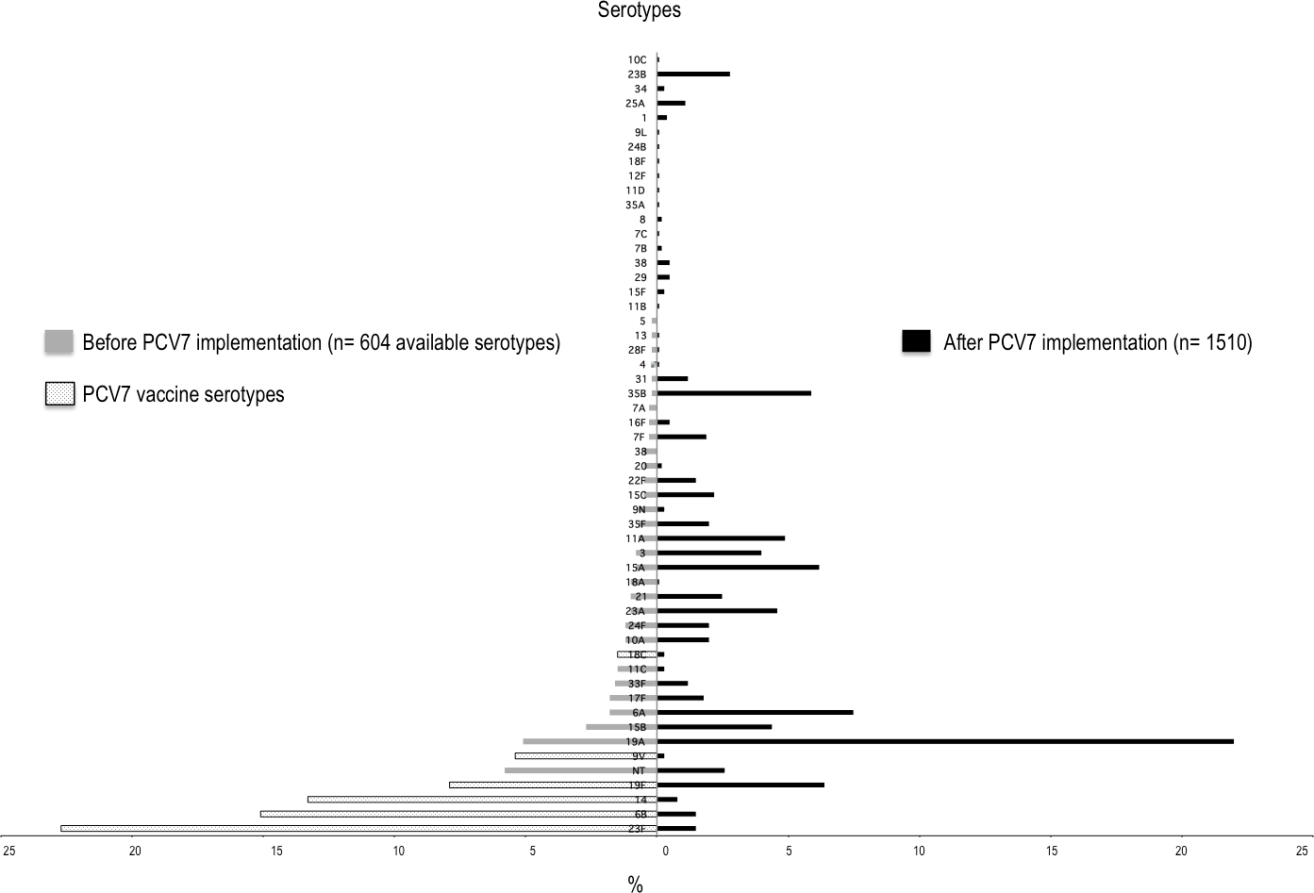
**Distribution of serotypes before and after 7-valent pneumococcal conjugate vaccine (PCV7) implementation**.

Table 2 presents the carriage rates of Sp, Hi, Mc and Sa before and after PCV7 implementation. Almost all Hi strains were non-typeable (98.5%). The carriage rates of Sp, NTHi and Sa did not differ between the 2 periods, but Mc carriage increased (5%). The Figure 1 presents the distribution of serotypes before and after PCV7 implementation. Among the Sp carriers, the proportion of PCV7 vaccine types (serotypes 4, 6B, 9 V, 14, 18 C, 19 F and 23 F) decreased from 66.6% to 10.7% (P < 0.001), penicillin intermediate-resistant strains increased from 30.3% to 43.4% (P < 0.001), and penicillin-resistant strains greatly decreased from 22.8% to 3.8% (P < 0.001). The proportion of Hi ß-lactamase-producing strains decreased from 38.6% to 17.1% (P < 0.001).

**Table 2:** Nasopharyngeal carriage of *Streptococcus pneumoniae, Haemophilus influenzae, Moraxella catarrhalis *and *Staphylococcus aureus *in children with AOM before and after PCV7 implementation

Carriage	Before PCV7 n = 1807 (%)	After PCV7 n = 2598 (%)	*p*-value*
*S. pneumoniae*	1047 (57.9)	1510 (58.1)	0.9

PCV7 vaccine types	402/604**(66.6)	162 (10.7)	0.0001

Penicillin intermediate- resistant	317 (30.3)	656 (43.4)	0.0001

Penicillin resistant	239 (22.8)	58 (3.8)	0.0001

Non-typable *H. influenzae*	849 (47)	1269 (48.8)	0.2

ß-lactamase+	328 (38.6)	217 (17.1)	0.0001

*M. catarrhalis*	939 (52)	1479 (56.9)	0.001

*S. aureus*	112 (6.2)	136 (5.2)	0.2

Multiple carriage (≥ 2 species)	978 (54.1)	1502 (57.8)	0.02

No carriage	133 (7.4)	187 (7.2)	0.8

* Chi square test

** on available serotypes

### Risk factors for carriage of S. pneumoniae, NT H. influenzae, M. catarrhalis, and S. aureus

We analyzed the factors influencing Sp, NTHI, Mc and Sa carriage by logistic regression analysis (Table 3) and found the following:

**Table 3:** Risk factors for carriage of *S. pneumoniae*, non-typable (NT) *H. influenzae, M. catarrhalis*, and *S. aureus *by univariate and multivariate analysis

	Univariate analysis		Multivariate analysis*	
	**OR [95% CI]**	**P value**	**OR [95% CI]**	**P value**

**Carriage of *S***. ***pneumoniae***				

Fever ≥ 38.5°C	1.47 1.30;1.66	< 0.0001	1.3 1.11;1.54	0.002

Daycare centre attendance	1.45 1.28;1.65	< 0.0001	1.51 1.28;1.78	< 0.0001

Carriage of *M. catarrhalis*	1.41 1.25;1.59	< 0.0001	1.17 1.01;1.36	0.034

Period of the study	1.01 0.89;1.14	0.905	0.85 0.69;1.05	0.130

Age ≥ 12 months	0.99 0.88;1.11	0.853	0.94 0.81;1.09	0.392

Antibiotic use 3 months before enrolment	0.80 0.69;0.92	0.002	0.76 0.66;0.89	< 0.0001

Carriage of *H. influenzae*	0.80 0.71;0.90	< 0.0001	0.84 0.72;0.98	0.028

Conjunctivitis	0.50 0.43;0.57	< 0.0001	0.52 0.44;0.62	< 0.0001

Carriage of *S. aureus*	0.36 0.27;0.46	< 0.0001	0.49 0.35;0.67	< 0.0001

**Carriage of NT *H. influenzae***				

Conjunctivitis	4.64 3.98;5.42	< 0.0001	4.39 3.67;5.26	< 0.0001

Daycare centre attendance	1.63 1.44;1.85	< 0.0001	1.40 1.18;1.65	< 0.0001

Age ≥ 12 months	1.43 1.27;1.62	< 0.0001	1.28 1.10;1.48	0.001

Antibiotic use 3 months before enrolment	1.27 1.10;1.46	0.001	1.22 1.05;1.42	0.01

Period of the study	1.08 0.96;1.22	0.224	0.89 0.73;1.08	0.261

Carriage of *S. pneumoniae*	0.80 0.71;0.90	< 0.0001	0.83 0.71;0.96	0.015

Carriage of *S. aureus*	0.47 0.35;0.62	< 0.0001	0.61 0.43;0.85	0.004

**Carriage of *M. catarrhalis***				

Daycare centre attendance	1.91 1.68;2.17	< 0.0001	1.83 1.60;2.09	< 0.0001

Carriage of *S. pneumoniae*	1.41 1.25;1.59	< 0.0001	1.23 1.08;1.39	0.001

Fever ≥ 38.5°C	1.30 1.15;1.46	< 0.0001	1.17 1.03;1.33	0.015

Period of the study	1.22 1.08;1.38	0.001	1.08 0.95;1.22	0.255

Age ≥ 12 months	1.14 1.01;1.28	0.037	1.05 0.93;1.19	0.424

Conjunctivitis	0.79 0.68;0.90	0.001	0.77 0.67;0.89	< 0.0001

Carriage of S. aureus	0.40 0.31;0.52	< 0.0001	0.44 0.33;0.58	< 0.0001

**Carriage of *S. aureus***				

Carriage of *S. pneumoniae*	0.36 0.27;0.47	< 0.0001	0.35 0.26;0.46	< 0.0001

Carriage of *H. influenzae*	0.47 0.35;0.62	< 0.0001	0.43 0.33;0.57	< 0.0001

Carriage of *M. catarrhalis*	0.40 0.31;0.52	< 0.0001	0.42 0.32;0.55	< 0.0001

Age ≥ 12 months	0.61 0.47;0.79	< 0.0001	0.65 0.50;0.85	0.002

Period of the study	0.84 0.65;1.08	0.173	0.92 0.70; 1.19	0.521

OR = odds ratio; 95% CI = 95% confidence interval.

*Tested variables in the regression logistic model were: period of the study, age, daycare attendances modalities, conjunctivitis, fever ≥ 38.5°C, otalgia, antibiotic 3 months before inclusion, Sp, Hi, Bc and Sa carriage. Only variables kept in the final model are presented in the table.

- Daycare center attendance was associated with increased carriage rates of Sp, NTHi and Mc.

- Age > 12 months was associated with increased NTHi carriage and with decreased Sa carriage.

- Fever ≥ 38.5°C was associated with an increased risk of Sp carriage.

- Conjunctivitis was associated with an increased risk of NTHi carriage and a decreased risk of Sp carriage.

Among potential biologic interactions, Sa carriage was associated with a reduction of Sp, NTHi and Mc carriage. By contrast, Mc carriage was associated with an increased risk of Sp carriage (AOR = 1.23, 95% CI [1.08; 1.39]).

In comparing the pre- and post-PCV7 periods, we found a slight but non-significant decrease in carriage rates of pneumococcus (AOR = 0.85 [0.69;1.05]), *H. influenzae *(AOR = 0.89 [0.73;1.09]) and *S. aureus *(AOR = 0.92 [0.70;1.19]). By contrast, the carriage rate of *M. catarrhalis *increased slightly but not significantly between the 2 periods (AOR = 1.08 [0.95;1.2]).

## Discussion

In this study, we studied NP samples of children with AOM before and after PCV7 implementation in France and found a marked reduction in vaccine serotype carriage after PCV7 implementation.

However, the overall pneumococcal, *H. influenzae *and *S. aureus *carriage slightly decreased but non significantly. These results agree with those from 2 prospective studies comparing vaccinated and non-vaccinated populations, one enrolling HIV-infected and HIV-uninfected children 5 years after completion of immunization in South Africa [22] and the other a randomized controlled trial of the impact of 2 doses and 2 + 1 doses of PCV7 on carriage of Sp, Hi and Mc in normal children in The Netherlands [22,23]. Reduced-dose PCV7 schedules slightly reduced overall pneumococcal carriage but not carriage of Hi and Mc [22,23]. The vaccination coverage reported in our study (> 98% in 2006-2009) is higher than that for all children in France (91%) [9]. However, the investigators in this study were well-trained pediatricians, and the vaccination coverage is higher in France for children followed by pediatricians than general practitioners.

Post-licensure studies of middle ear fluid of patients with AOM after PCV7 implementation in the United States have shown a relative increase in *H. influenzae*- and *M. catarrhalis-*positive middle-ear fluid cultures, together with decreased pneumococcal and vaccine serotype AOM. These were ecological and uncontrolled observations and do not necessarily represent true increases in otitis media due to non-pneumococcal pathogens [24]. The reduction of ß-lactamase-producing NTHi strains is probably due to the overall reduction in antibiotic prescriptions in France with the Health Authorities promotional campaign for appropriate antibiotic prescription [25,26]. In fact, the most prescribed antibiotics for AOM (leading reason for antibiotic prescriptions for infants and toddlers) were cefpodoxime proxetil and amoxicillin-clavulanate [25]. Under these selective pressures, ß-lactamase-producing NTHi strains have no ecological advantage. Of note, these regimens could lead to increased ß-lactamase-negative ampicillin-resistance strains in France [27].

Our study has several limitations. The dataset used for the pre-PCV7 period combined the results of 4 different studies performed during 7 years. Because of the relative heterogeneity of the results, several epidemiological and clinical characteristics (daycare attendance, antibiotics, fever or otalgia) that might influence carriage rates significantly differed between the 2 periods, which necessitated multivariate analysis. Also data for some clinical and demographic features were missing in the pre-PCV7 period. Finally, data that may also influence carriage rates, such as crowding or siblings, were not recorded before and after PCV7 implementation.

Recently available pneumococcal conjugate vaccines with broader serotype coverage may lead to reduced overall pneumococcal carriage; the impact on the carriage of other NP pathogens requires on-going monitoring.

## Conclusions

After implementation of PCV7 vaccine in France, no significant changes in NP carriage with *S. pneumoniae, H. influenzae, M. catarrhalis *and *S. aureus *were observed in children with AOM. Effects over time with high-vaccine pressure following nationwide implementation of pneumococcal conjugate vaccines with broader serotype coverage need to be followed.

## Competing interests

The authors declare that they have no competing interests.

## Authors' contributions

RC designed the study and drafted the manuscript. CL coordinated the participating centers, was responsible for the statistical analysis and drafted the manuscript. EB helped draft the manuscript. FT and VD were the main investigators and helped draft the manuscript. MB designed the database and helped draft the manuscript. EB and EV performed bacteriological analyses and helped draft the manuscript. All authors read and approved the final manuscript.

## Authors' information

Robert Cohen is a French pediatric infectious disease specialist, scientific director of a research institute on pediatric community acquired infections (ACTIV) and scientific director of a vaccine network for healthcare workers (Infovac-France). His main research interests are epidemiologic studies and clinical trials in community acquired infections, including pneumococcal diseases, rhinopharyngeal flora, and vaccines. He has published more than 45 papers in English.

## Pre-publication history

The pre-publication history for this paper can be accessed here:

http://www.biomedcentral.com/1471-2334/12/52/prepub
